# Higher Cerebral Blood Flow Predicts Early Hematoma Expansion in Patients With Intracerebral Hemorrhage: A Clinical Study

**DOI:** 10.3389/fneur.2021.735771

**Published:** 2021-12-06

**Authors:** Weijing Wang, Weitao Jin, Hao Feng, Guoliang Wu, Wenjuan Wang, Jiaokun Jia, Ruijun Ji, Anxin Wang, Xingquan Zhao

**Affiliations:** ^1^Department of Neurology, Beijing Tiantan Hospital, Capital Medical University, Beijing, China; ^2^Department of Neurology, Beijing Haidian Hospital, Beijing, China; ^3^Department of Neurology, Haidian Section of Peking University Third Hospital, Beijing, China; ^4^Department of Neurosurgery, Peking University International Hospital, Beijing, China; ^5^China National Clinical Research Center for Neurological Diseases, Beijing, China; ^6^Research Unit of Artificial Intelligence in Cerebrovascular Disease, Chinese Academy of Medical Sciences, Beijing, China; ^7^Department of Radiology, Beijing Tiantan Hospital, Capital Medical University, Beijing, China

**Keywords:** intracerebral hemorrhage, hematoma expansion, cerebral blood flow (CBF), CT perfusion (CTP), prognosis

## Abstract

The early hematoma expansion of intracerebral hemorrhage (ICH) indicates a poor prognosis. This paper studies the relationship between cerebral blood flow (CBF) around the hematoma and hematoma expansion (HE) in the acute stage of intracerebral hemorrhage. A total of 50 patients with supratentorial cerebral hemorrhage were enrolled in this study. They underwent baseline whole-brain CTP within 6 h after intracerebral hemorrhage, and non-contrast CT within 24 h. Absolute hematoma growth and relative hematoma growth were calculated, respectively. A relative growth of Hematoma volume >33% was considered to be hematoma expansion. The Ipsilateral peri-edema CBF and Ipsilateral edema CBF were calculated by CTP maps in patients with and without hematoma expansion, respectively. In this study the incidence of hematoma expansion in the early stage of supratentorial cerebral hemorrhage was 32%; The CBF of the hematoma expansion group was higher than that of the patients without hematoma expansion (23.5 ± 12.5 vs. 15.1 ± 7.4, *P* = 0.004). After adjusting for age, gender, Symptom onset to NCCT and Baseline hematoma volume, ipsilateral peri-edema CBF was still an independent risk factor for early HE (or = 1.095, 95% CI = 1.01–1.19, *P* = 0.024). Here, we concluded that higher cerebral blood flow predicts early hematoma expansion in patients with intracerebral hemorrhage.

## Introduction

Acute spontaneous (non-traumatic) intracerebral hemorrhage is the most common type of spontaneous intracerebral hemorrhage. It affects about 2 million people around the world every year and exhibits the worst prognosis of all stroke types (1). The burden of hemorrhagic stroke is increasing rapidly worldwide between 1990 and 2010, with an increase of 47% in the absolute number of people with incident hemorrhagic stroke, 20% in deaths, and 14% in DALYs (1).

Intracerebral hemorrhage (ICH) expansion occurs in about one-third of ICH patients and is strongly associated with a poor outcome (2, 3).

Several imaging signs on non-contrast CT may be related to the hematoma expansion and poor prognosis after intracerebral hemorrhage (4–8). The spot sign on CT angiography also received attention as an indicator of the hematoma expansion (9). However, the interpretation of those signs in the CT relies on the clinical experience of neuroradiologists, and the standard of that interpretation is not objective and cannot be evaluated quantitatively.

In our study, CBF was used as a quantitative method to calculate the cerebral blood flow of edematous tissue around a hematoma and its surrounding tissue after early hemorrhage, so as to study the correlation between the cerebral blood flow and the hematoma expansion.

## Methods

### Study Design and Population Eligibility

**Figure 1:**
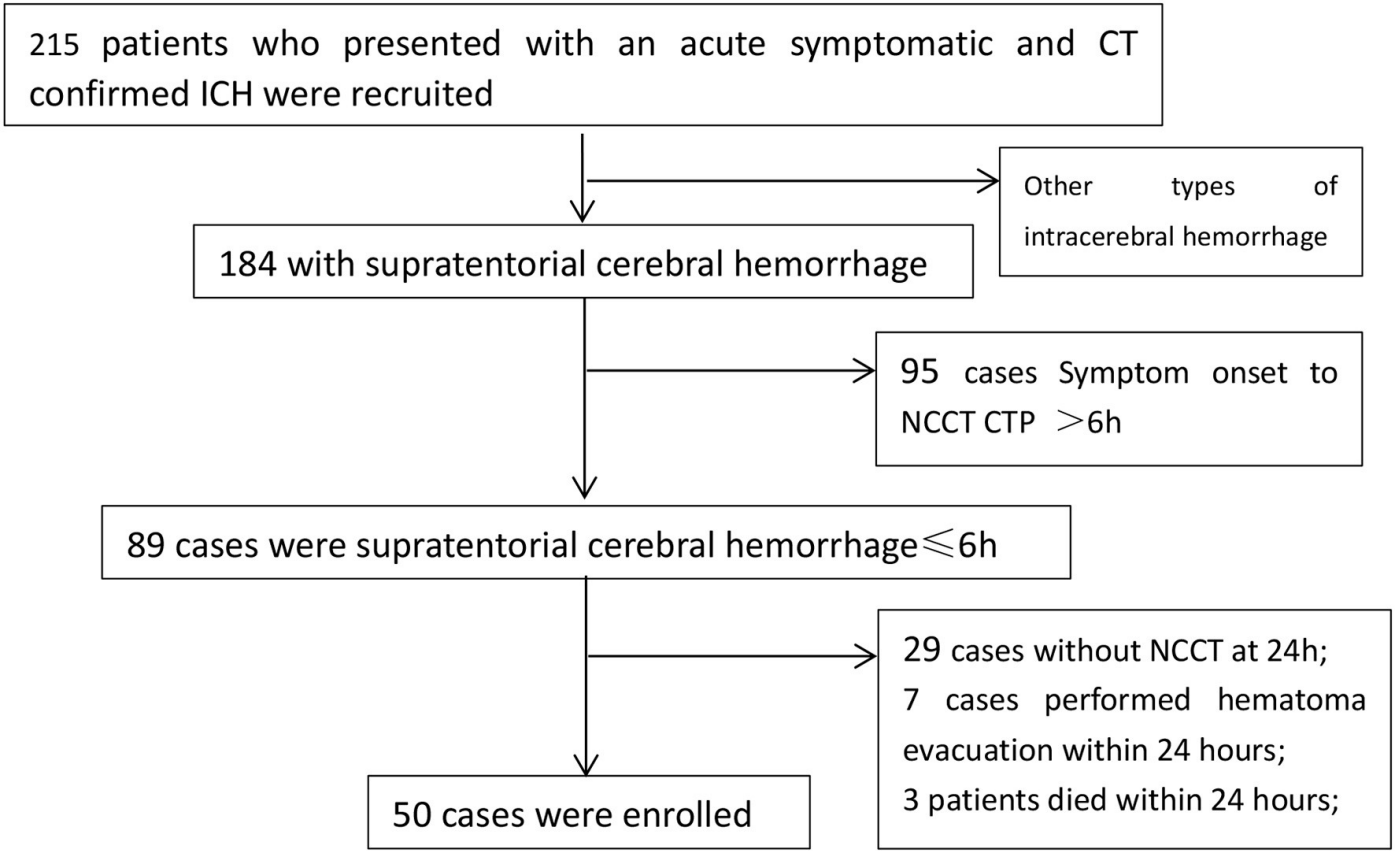
Flow chart of patient selection.

This study was a prospective, observational cohort study. From December 2014 to September 2016 patients who presented with an acute symptomatic and CT confirmed ICH were recruited to the study. Patients aged 18 years or older were eligible for entry. NCCT was performed in 215 patients who had acute symptoms (severe headache, paralysis, aphasia etc.). 184 patients were confirmed supratentorial intracerebral hemorrhage. 50 of them finished CTP within 6 hours and NCCT review within 24 hours after the symptoms attack. Therefore, 50 patients are included in this study (Figure 1).

Inclusion criteria: (1) age > 18 years, (2) within 6 h of onset, (3) NCCT + CTA + CTP at baseline, (4) NCCT reexamination within 24 h of onset, (5) informed consent. Exclusion criteria: (1) failure to cooperate with the completion of imaging examination (refusal of examination or sensitization of imaging agent), (2) planned or completed surgical craniotomy or hematoma aspiration, (3) refusal to join the study.

### Sources of Funding

This study was sponsored by Capital health research and development of special (grant number: Capital 2011–2004-03; institute: Beijing Municipal Commission of Health and Family Planning; the author who received the funding: XZ) and the Beijing Municipal Science & Technology Commission, PR China (grant number: Z131107002213009).

### Image Acquisition

All participants were scanned on a 16-slice multidetector CT (Somatom volume zoom; Siemens, Erlangen, Germany). Whole-brain NCCT was performed first with a slice thickness of 9 mm for the supratentorial area and 4.5 mm for the infratentorial area to confirm primary ICH. Acquisition parameters were: 120 kVp; 310 mA; and field of view (FOV) = 24 cm (10).

CTP covering two continuous sections at the level across the maximum transverse section of the hematoma lesion was performed. The scanning parameters were: tube current = 80 kVp; 209 mA; rotation time¼ 1.0 s/rotation; total scan time 40 s; section thickness = 12 mm; and 40 images per section. CTP was started 4 s after injection of a bolus of 40 ml of iobitridol (300 mg/mL, Xenetix; Guerbet, Aulnay-sous-Bois, France) at a rate of 8 mL/s into the antecubital vein (with a 20-gauge intravenous cannula) using a power injector. The effective radiation dose was 3.51 mSv for one-time scanning (10).

All patients were followed up with NCCT using the same CT system and parameters 24 h after the onset of the disease to evaluate whether the hematoma expanded.

### Image Analysis

Hematoma volumes were calculated at baseline and at follow-up NCCT images using the method as follows: Hematoma volumes were defined using semiautomated Hounsfield Unit thresholding. Edema volumes were measured using a threshold of 5 to 23 Hounsfield Units, which has been demonstrated to be the most reliable CT Hounsfield Unit threshold for edema (11, 12).

**Figure 2:**
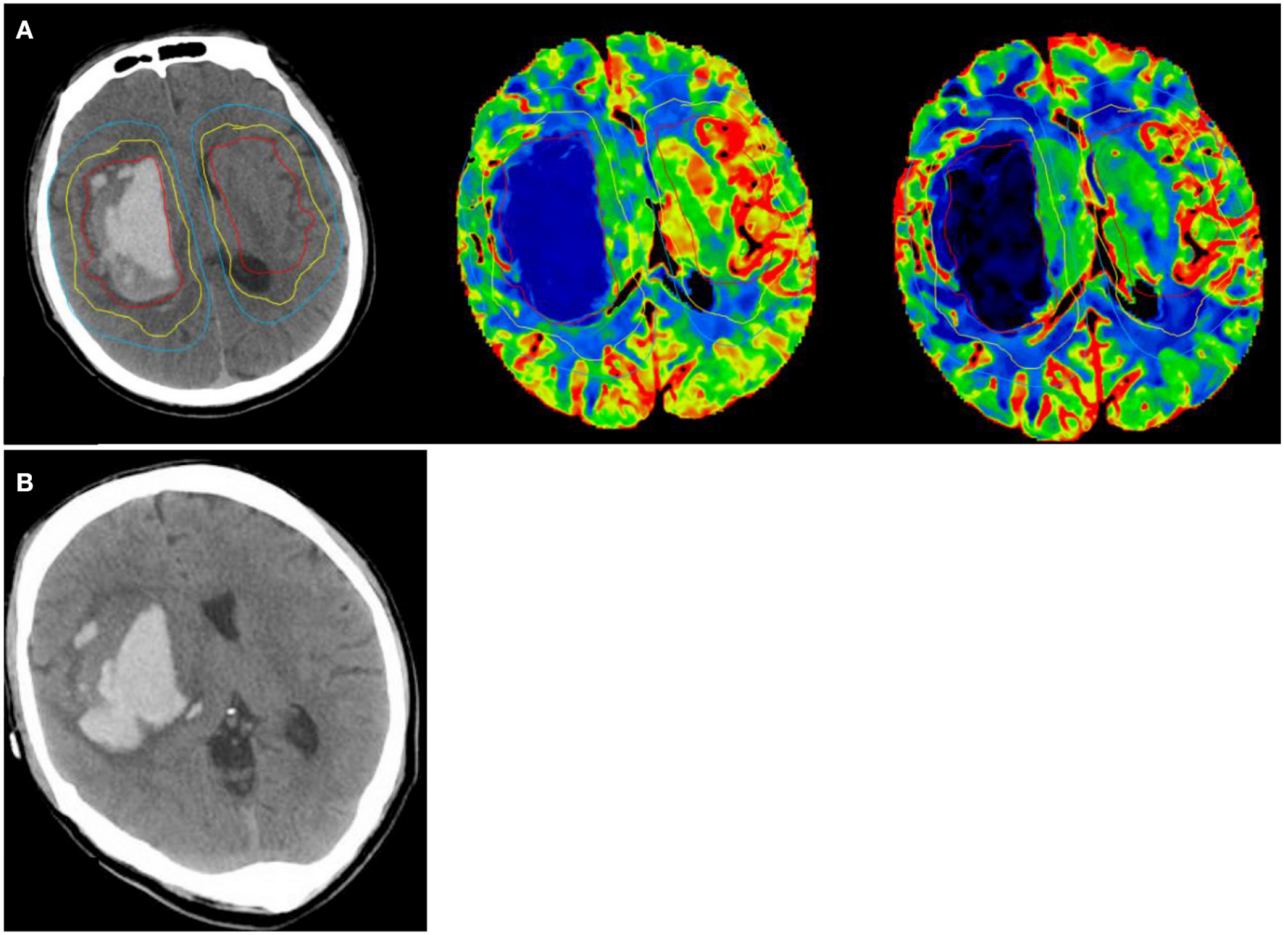
A 42 year old man with a sudden headache and loss of consciousness, NCCT shows hematoma volume 49.5 ml at randomization. Ipsilateral peri-edema CBF was 30.48 ml/100 g/min at randomization. **(A)** (1) perihematomal low-density area in the yellow circle as Ipsilateral edema area; (2) an area mirroring the Ipsilateral edema area located in the contralateral hemisphere as Contralateral edema area; (3) 1 cm rim of normal-appearing brain tissue surrounding the perilesional low-density area in the blue circle as Ipsilateral peri-edema area; (4) an area mirroring the Ipsilateral peri-edema area located in the contralateral hemisphere as Contralateral peri-edema area. **(B)** NCCT at 24 h after symptom onset shows hematoma volume was 69.14 ml.

Absolute ICH growth (follow-up volume–baseline volume) and relative ICH growth ([follow-up volume–baseline volume]/baseline volume) were calculated, respectively. A relative hematoma increase growth >33% was considered to indicate significant hematoma expansion (13). Post-processing of raw CTP source images was completed centrally on a GE aw workstation. CTP maps were derived from the tissue time–density curve and contrast bolus delay and dispersion were corrected for using a singular value deconvolution algorithm. Region of interest (ROI) analyses were completed using the Analyze 11.0 software package (13). As previously reported (14), ROIs were drawn using planimetric techniques on CTP base images and then transferred to the corresponding CBF, CBV, MTT maps. ROIs included the edema, a 1-cm region surrounding the edema, contralateral mirror regions (Figure 2). Image post-processing was completed by an experienced neuroradiologist and then reviewed by a chief radiologist independently.

### Statistical Analysis

Statistical analysis was performed using SPSS 21.0 (SPSS Inc, Chicago, IL). Comparison of baseline mean characteristics between the 2 groups was made using independent *t*-tests, Mann–Whitney tests, or Pearson χ^2^-tests. The relationships between hematoma expansion and CBF were assessed using Logistic regression. Paired *t*-tests were used to assess CBF differences between different parts. The influence of different sites and CBF on hematoma expansion was evaluated by the method of interaction analysis.

## Results

### Baseline Characteristics

A total of 50 patients were included in the study (Baseline characteristics shown in Table 1). The study population included 34 (68.0%) males and 16 (32.0%) females with an average age of 50.8 years. The baseline demographic and clinical cohort characteristics are described in Table 1. The median time from symptom onset to baseline CT scan was 3.6 ± 1.5 h, The median baseline hematoma volume was 24.7 ± 22.0 ml. 16 patients (32%) showed early hematoma expansion. All patients completed CT Perfusion at baseline. There was a difference between the two groups in the CBF of the peri-edema area. (CBF on the peri-edema area of patients with hematoma expansion was 23.5 ml/100 g/min; CBF on the peri-edema area of patients without hematoma expansion was 15.1 ml/100 g/min; *p*-value 0.004).

**Table 1:** Baseline characteristics.

	**Total *N* = 50**	**Without hematoma** **expansion, *N* = 34**	**With hematoma** expansion^*^, *N* = 16	***p***
Age, mean ± SD	50.8 ± 13.4	51.9 ± 2.3	48.6 ± 3.50	0.42
Gender, Male, *n* (%)	34 (68.0)	22 (64.7)	12 (75.0)	0.47
Time from symptom onset to randomization, h, mean ± SD	3.3 ± 1.6	3.2 ± 1.6	3.4 ± 1.6	0.72
Time from symptom onset to NCCT, h, mean ± SD	3.6 ± 1.5	3.5 ± 0.3	3.9 ± 0.4	0.48
**Medical history**				
Hypertension, *n* (%)	29 (58.0)	1 (55.9)	10 (62.5)	0.66
Diabetes, *n* (%)	9 (18.0)	6 (17.7)	3 (18.8)	0.93
Ischemic stroke, *n* (%)	5 (10.0)	3 (8.8)	2 (12.5)	0.69
Previous ICH, *n* (%)	3 (6.0)	2 (5.9)	1 (6.3)	0.96
**Clinical characteristics**				
mRS score before onset, ≤1, *n* (%)	49 (98.0)	34 (100.0)	15 (93.8)	0.18
BMI, median (IQR)	24.6 (22.7–26.8)	25.3 (22.8–27.6)	24.2 (22.3–26.1)	0.35
Systolic BP, mmHg, mean ± SD	169.8 ± 23.8	166.1 ± 4.3	177.5 ± 5.2	0.12
Diastolic BP, mmHg, mean ± SD	99.7 ± 16.6	97.2 ± 2.8	104.7 ± 4.1	0.14
Heart rate, bpm, mean ± SD	77.8 ± 11.2	76.1 ± 11.8	81.1 ± 9.3	0.16
NIHSS score, mean ± SD	9.1 ± 6.3	9.1 ± 1.1	9.2 ± 1.5	0.96
GCS score, median (IQR)	14.0 (13.0–15.0)	14.0 (12.8–15.0)	14.0 (14.0–15.0)	0.33
**Hematoma**				
Lobar, *n* (%)	12 (24.0)	8 (23.5)	4 (25.0)	0.91
Deep, *n* (%)	38 (76.0)	26 (76.5)	12 (75.0)	0.91
Baseline hematoma volume, ml, mean ± SD	24.7 ± 22.0	26.3 ± 22.6	21.5 ± 21.2	0.48
24 h-Hematoma volume, ml, median (IQR)	24.6 (9.1–45.7)	21.1 (8.5–41.9)	29.81 (10.4–52.8)	0.28
**Edema**				
Baseline edema volume, ml, median (IQR)	15.2 (9.3–23.9)	14.0 (7.4–23.9)	16.8 (10.0–24.9)	0.51
24 h-Edema volume, ml, median (IQR)	20.2 (10.6–31.3)	19.9 (11.8–31.3)	20.2 (10.0–32.1)	0.97
**Cerebral blood flow**				
Ipsilateral edema CBF, ml/100 g/min, mean ± SD	11.8 ± 6.6	12.3 ± 6.8	10.7 ± 6.3	0.42
Ipsilateral peri-edema CBF, ml/100 g/min, mean ± SD	17.8 ± 10.0	15.1 ± 7.4	23.5 ± 12.5	0.004
**Cerebral blood volume**				
Ipsilateral edema CBV, ml/100 g, mean ± SD	1.5 ± 0.7	1.5 ± 0.7	1.4 ± 0.7	0.60
Ipsilateral peri-edema CBV, ml/100 g, mean ± SD	1.9 ± 0.9	1.8 ± 0.9	2.1 ± 0.8	0.17
**Mean transit time**				
Ipsilateral edema MTT, s, mean ± SD	10.4 ± 3.4	10.3 ± 3.5	10.6 ± 3.5	0.80
Ipsilateral peri-edema MTT, s, mean ± SD	8.6 ± 2.8	9.1 ± 2.8	7.6 ± 2.7	0.095
**Laboratory**				
WBC, 10 ^∧^ 9/L, mean ± SD	9.4 ± 3.8	9.6 ± 3.7	9.1 ± 3.9	0.67
PLT, 10 ^∧^ 9/L, mean ± SD	218.1 ± 56.6	223.9 ± 10.6	205.6 ± 10.7	0.29
INR, mean ± SD	0.9 ± 0.1	0.9 ± 0.0	0.9 ± 0.0	0.68
Fbg, ug/ml, mean ± SD	2.5 ± 0.6	2.6 ± 0.5	2.5 ± 0.8	0.47
APTT, sec, mean ± SD	26.2 ± 3.9	25.8 ± 3.4	26.9 ± 4.9	0.39
Glu, mmol/L, mean ± SD	7.5 ± 2.6	7.6 ± 0.5	7.3 ± 0.6	0.68
Crea, umol/L, mean ± SD	61.2 ± 10.9	62.1 ± 2.0	59.7 ± 2.6	0.52
Bun, mmol/L, mean ± SD	5.1 ± 1.4	5.2 ± 1.2	4.9 ± 1.7	0.58
Total cholesterol, mmol/L, mean ± SD	4.7 ± 1.0	4.5 ± 1.0	4.9 ± 1.0	0.34
Triglyceride, mmol/L, mean ± SD	1.5 ± 1.3	1.5 ± 1.5	1.6 ± 0.4	0.84
HDL, mmol/L, mean ± SD	1.3 ± 0.4	1.3 ± 0.5	1.3 ± 0.4	0.97
LDL, mmol/L, mean ± SD	2.8 ± 0.9	2.8 ± 0.9	2.9 ± 0.9	0.63
**Clinical outcomes**				
NIHSS 24, mean ± SD	8.5 ± 6.1	8.6 ± 1.0	8.2 ± 1.5	0.83
GCS 24, median (IQR)	14.0 (14.0–15.0)	14.0 (12.8–15.0)	14.5 (14.0–15.0)	0.44

* *Hematoma expansion defined as an absolute increase in hematoma >12.5 ml or relative growth >33%*.

### CBF in Different Locations

The mean Ipsilateral edema CBF was 11.8 ± 6.6 ml/100 g/min. The mean Ipsilateral peri-edema CBF was 17.8 ± 10.0 ml/100 g/min. The Ipsilateral peri-edema CBF was higher than Ipsilateral edema CBF (17.8 ± 10.0 ml/100 g/min vs. 11.8 ± 6.6 ml/100 g/min, *p* = 0.001) in all groups, the Ipsilateral peri-edema CBF was higher than Ipsilateral edema CBF (15.1 ± 7.4 ml/100 g/min vs. 12.3 ± 6.8 ml/100 g/min, *p* = 0.099) in patients without hematoma expansion group, and the Ipsilateral peri-edema CBF was higher than Ipsilateral edema CBF (23.5 ± 12.5 ml/100 g/min vs. 10.7 ± 6.3 ml/100 g/min, *p* = 0.099) in patients with hematoma expansion group (Table 2).

**Table 2:** CBF in different location.

	**Ipsilateral edema CBF, ml/100 g/min**	**Ipsilateral peri-edema CBF, ml/100 g/min**	***p***
Total, *n* = 50	11.80 ± 6.64	17.83 ± 10.01	0.001
Without hematoma expansion, *n* = 34	12.33 ± 6.81	15.14 ± 7.40	0.099
With hematoma expansion^*^, *n* = 16	10.67 ± 6.33	23.54 ± 12.48	0.002

* *Hematoma expansion defined as an relative increase in hematoma >33%*.

### Multivariate Analysis of Hematoma Expansion

Univariate analysis showed that Ipsilateral peri-edema CBF was a risk factor for early hematoma expansion (OR = 1.10, 95% CI 1.02–1.19, *P* = 0.014). Model 1 included covariates with *p* < 0.20 in univariable analysis. Model 2 adjusted for age and sex. Model 3 adjusted for age, sex, and predictors of hemorrhage growth identified from the literature such as time from onset to NCCT and ICH volume. Multivariate analysis showed that Ipsilateral peri-edema CBF was an independent risk factor for hematoma expansion (Table 3).

**Table 3:** Multivariate analysis of hematoma expansion.

		**OR**	**OR (95% CI)**	***p***
Model 1	SBP	1.009	0.971–1.048	0.655
	DBP	1.031	0.973–1.092	0.303
	Ipsilateral peri-edema CBF	1.101	1.022–1.185	0.011
Model 2	SBP	1.013	0.973–1.054	0.541
	DBP	1.030	0.971–1.092	0.330
	Ipsilateral peri-edema CBF	1.260	1.022–1.544	0.031
	Ipsilateral peri-edema CBV	0.288	0.050–1.661	0.164
	Ipsilateral peri-edema MTT	1.336	0.859–2.079	0.199
Model 3	Age	1.010	0.953–1.070	0.749
	Gender (male)	2.569	0.446–14.799	0.291
	SBP	1.011	0.970–1.054	0.595
	DBP	1.033	0.972–1.099	0.296
	Ipsilateral peri-edema CBF	1.091	1.011–1.178	0.025
Model 4	Age	1.010	0.949–1.075	0.754
	Gender (male)	4.447	0.556–35.543	0.159
	SBP	1.017	0.974–1.061	0.452
	DBP	1.032	0.970–1.098	0.315
	Ipsilateral peri-edema CBF	1.095	1.012–1.185	0.024
	Contralateral peri-edema CBF	1.031	0.949–1.120	0.476
	Symptom onset to NCCT (h)	1.293	0.754–2.216	0.350
	Baseline hematoma volume (ml)	0.983	0.945–1.021	0.370

*Model 1: unadjusted*.

*Model 2: Adjusted CBV and MTT*.

*Model 3: Adjusted age and gender*.

*Model 4: Adjusted age, gender, Symptom onset to NCCT, and Baseline hematoma volume*.

## Discussion

The incidence of hematoma expansion in the early stage of supratentorial intracerebral hemorrhage is 32% in this study. Hematoma expansion was defined as a 33% increase in hematoma volume compared with baseline (13), which is consistent with the results Brott et al. (15) reported in the previous literature of about 38%. However, due to the disunity of the definitional standard of hematoma expansion, the incidence of hematoma expansion reported in the relevant studies is quite different. The longer the difference between the first CT and the reexamination CT is, the closer the first CT is to the onset time, and the incidence of hematoma expansion is correspondingly increased. In 1990, Fujitsu et al. (16) studied 107 patients with intracerebral hemorrhage within 6 h of onset, rescanned NCCT within 24 h, and found that the incidence of hematoma expansion was about 20.3%; Mayer et al. (17) observed 46 patients with intracerebral hemorrhage within 11 h of onset, and the hematoma expansion rate was only 9%.

In recent years, some clinical studies have focused on the relationship between hematoma expansion and prognosis. Some results have revealed the relationship between hematoma expansion and poor prognosis. Davis et al. (18) in a 2006 meta-analysis showed that hematoma volume increased by 10%, mortality increased by 5%.

However, the pathophysiological mechanism of hematoma expansion is still unclear. Further study on the pathophysiological mechanism of hematoma expansion and the cerebral blood flow around the hematoma is helpful to clarify the causes of hematoma expansion, so as to provide targeted prevention and treatment and improve the prognosis.

This study showed the CBF of ipsilateral peri-edema was higher than that of edema CBF, suggesting that after cerebral hemorrhage, the edema was hypoperfusion, and there was an area of hyperperfusion around the edema. Using single-photon emission computed tomography (SPECT), Mayer et al. (19) demonstrated depressed perfusion surrounding the hemorrhage and concluded that the restoration of perilesional blood flow in the early stage of ICH resulted from perihematoma cerebral edema formation. Zhou et al. (20) found that a gradient of hypoperfusion surrounding the hematoma, which showed a thin rim of reduced perfusion in the perilesional zone in rCBF maps. In addition to perilesional hyperperfusion, the local hyperemia can also be observed in the regions distant from the hematoma, even in the cortical regions of the uninvolved hemisphere. The hyperemia often results from the vasodilation of pia arteries and arterioles in the periphery of the injury zone (19), as efforts are made to restore blood flow in the regions of reduced perfusion after ICH onset. The unstable blood flow perfusion occurring in perilesional tissue and remote brain may, on the one hand, reflect the exhaustion of autoregulation of CBF correlated with ICH, and on the other hand, predict that the secondary brain injury will occur in the related cerebral tissue.

Intracerebral hemorrhage is a dynamic, complex, and continuous process. Hematoma expansion occurs in the early stage of intracerebral hemorrhage. The hematoma expansion comes from blood pressure. The higher the blood pressure is, the more likely the hematoma to expand. There are two reasons why the hematoma is no longer enlarged. One is the spontaneous hemostasis mechanism after vascular rupture; the other is that the pressure inside and outside the ruptured blood vessel wall is reduced due to the pressure increase in the hematoma cavity caused by edema compression. Therefore, the study of edema area and surrounding tissue is helpful to understand the pathophysiological mechanism of hematoma expansion.

On the other hand, some imaging signs can also indicate the occurrence of hematoma expansion. The most classic is the “spot sign” (9). A spot sign is defined as one or more 1- to 2-mm foci of enhancement within the hematoma on CTA source images, which is the exudation point of contrast inside the hematoma. The occurrence of spot signs indicates an increased risk of hematoma expansion. The spot signs indicated that the enlargement of the hematoma was caused by the internal part of the hematoma.

However, some researchers have found that island sign can also indicate the risk of hematoma expansion (8). Island sign is the irregular edge of hematoma, suggesting the occurrence of hematoma expansion, which is caused by the fact that some relatively normal tissues outside the hematoma also become hemorrhagic brain tissue.

This study showed that there were blood flow changes in the peripheral tissue of hematoma. The higher the CBF of the tissue at the far side of the edema, the more likely the hematoma to expand, suggesting that the hematoma expansion may start from the outside.

However, the exact pathophysiological mechanism of hematoma expansion remains to be confirmed, for patients with ICH who have completed CTP examination in the early stage of admission, the measurement of ipsilateral peri edema CBF can be used as a supplementary prediction tool.

## Study Limitation

In our study, due to the strict limitations of timing and sequence of imaging examination, only 50 patients met the inclusion criteria, and more than 150 patients were excluded, which may have resulted in a selective bias. On the other hand, the patients who underwent NCCT and CTP at baseline and reviewed NCCT 24 h usually need to be in a relatively stable condition, and so for the patients with a large or deadly hemorrhage, whether the conclusion is still applicable is not clear. This study found that the relative increased rCBF between the peri-hematoma region and the contralateral region related with the hemorrhage expansion, but the increase of the CBF in peri-hematoma region did not show a similar result. This could be due to an interference caused by the treatments such as lowering the blood pressure or use of mannitol. These treatments could reduce the CBF of both sides, but the different vascular reactivity between the tissues surrounding the hematoma and the contralateral side made the range of decrease different, and then impacted on the result. However, this assumption still requires further study to be confirmed.

## Conclusion

Higher cerebral blood flow predicts early hematoma expansion in patients with intracerebral hemorrhage.

## Data Availability Statement

The original contributions presented in the study are included in the article/supplementary material, further inquiries can be directed to the corresponding author/s.

## Ethics Statement

The studies involving human participants were reviewed and approved by Ethics Committee of Tiantan Hospital. The patients/participants provided their written informed consent to participate in this study.

## Author Contributions

WeiW and XZ contributed to the conception and design of the study. WenW and JJ organized the database. HF and GW performed the statistical analysis. WeiW and WJ wrote the first draft of the manuscript. RJ and AW revised the manuscript. All authors contributed to manuscript revision, read, and approved the submitted version.

## Funding

This study was sponsored by Capital Health Research and Development of Special (Grant No. Capital 2011-2004-03; Institute: Beijing Municipal Commission of Health and Family Planning; the author who received the funding: XZ) and the Beijing Municipal Science & Technology Commission, PR China (Grant No. Z131107002213009).

## Conflict of Interest

The authors declare that the research was conducted in the absence of any commercial or financial relationships that could be construed as a potential conflict of interest.

## Publisher's Note

All claims expressed in this article are solely those of the authors and do not necessarily represent those of their affiliated organizations, or those of the publisher, the editors and the reviewers. Any product that may be evaluated in this article, or claim that may be made by its manufacturer, is not guaranteed or endorsed by the publisher.
